# Kyste mésothélial simple simulant un kyste hydatique

**DOI:** 10.11604/pamj.2014.17.314.4369

**Published:** 2014-04-25

**Authors:** Tarik Souiki, Karim Ibn Majdoub Hassani

**Affiliations:** 1 Service de Chirurgie Viscérale, CHU Hassan II, Fès, Maroc

**Keywords:** Kyste mésothélial, kyste hydatique, diagnostic différentiel, Kmesothelial system, hydatid cyst, differential diagnosis

## Image en medicine

Les kystes mésothéliaux simples sont rares et bénins. Leur pathogénie reste mal élucidée; Il semble qu'ils sont du à un défaut congénital d'accolement des surfaces péritonéales. Souvent pauci-symptomatiques, Leur découverte peut se faire ainsi fortuitement à l'imagerie, posant alors un problème de diagnostic différentiel par rapport aux autres formations kystiques péritonéales. Seul l'examen anatomopathologique de la pièce opératoire permet de confirmer le diagnostic. Nous rapportons l'observation d'une patiente âgée de 55 ans, sans antécédents notables, admise dans notre service pour prise en charge d'un kyste hydatique du foie: douleur de l'hypochondre droit et image typique à l’échographie d'un kyste hydatique de type 2 (classification de Gharbi) de 14 cm de diamètre, occupant le lobe droit hépatique. Le scanner abdominal réalisé pour une meilleure caractérisation topographique de ce kyste hydatique met en évidence, en plus de ce dernier, une image kystique à contenu liquidien et à paroi non rehaussée après injection de produit de contraste. Elle occupe la région rétro-péritonéale médiane et mesure 9 cm au grand axe (A,B). Vu le contexte clinique, ce kyste aspécifique a été considéré initialement comme une seconde localisation hydatique. L'intervention chirurgicale a été conduite par voie sous costale droite. Un traitement conservateur du KH hépatique a été réalisé dans un premier temps. Ensuite, un décollement colo-pariétal droit fait découvrir un kyste rétro-péritonéal à paroi mince translucide et à contenu séreux (C). Une énucléation de ce kyste a été réalisée. L'examen anatomopathologique porte le diagnostic de kyste mésothélial simple. Le pronostic de cette pathologie bénigne reste excellent avec un risque de récidive nul après exérèse chirurgicale complète.

**Figure 1:**
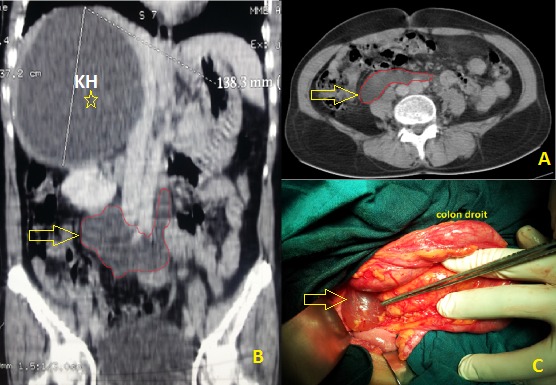
A) Coupe scanographique abdominale (L3): montrant une formation kystique d'environ 9 cm de grand axe occupant la région rétro-péritonéale médiane; B) Reconstruction scanographique montrant le kyste hydatique du foie et la formation kystique rétro-péritonéal; C) Vue opératoire du kyste mésothélial rétro-péritonéal après décollement colo-pariétal droit

